# Characterization of a bacterial strain *Lactobacillus paracasei* LP10266 recovered from an endocarditis patient in Shandong, China

**DOI:** 10.1186/s12866-021-02253-8

**Published:** 2021-06-17

**Authors:** Qi Tang, Yingying Hao, Lu Wang, Chao Lu, Ming Li, Zaifeng Si, Xiaoben Wu, Zhiming Lu

**Affiliations:** 1 Department of Clinical Laboratory, Shandong Provincial Hospital, Cheeloo College of Medicine, Shandong University, Jinan, 250021 Shandong China; 2 Department of Clinical Laboratory, Shandong Provincial Hospital Affiliated to Shandong First Medical University, Jinan, 250021 Shandong China; 3 Department of Dermatology, Qilu Hospital (Qingdao), Cheeloo College of Medicine, Shandong University, Qingdao, 266035 Shandong China; 4 Department of Clinical Laboratory, Dezhou Traditional Chinese Medicine Hospital, Dezhou, 253000 Shandong China

**Keywords:** *Lactobacillus paracasei* LP10266, Infective endocarditis, Genome sequencing, Biofilm

## Abstract

**Background:**

Lactobacilli are often recognized as beneficial partners in human microbial environments. However, lactobacilli also cause diseases in human, e.g. infective endocarditis (IE), septicaemia, rheumatic vascular disease, and dental caries. Therefore, the identification of potential pathogenic traits associated with lactobacilli will facilitate the prevention and treatment of the diseases caused by lactobacilli. Herein, we investigated the genomic traits and pathogenic potential of a novel bacterial strain *Lactobacillus paracasei* LP10266 which has caused a case of IE. We isolated *L. paracasei* LP10266 from an IE patient’s blood to perform high-throughput sequencing and compared the genome of strain LP10266 with those of closely related lactobacilli to determine genes associated with its infectivity. We performed the antimicrobial susceptibility testing on strain LP10266. We assessed its virulence by mouse lethality and serum bactericidal assays as well as its serum complement- and platelet-activating ability. The biofilm formation and adherence of strain LP10266 were also studied.

**Results:**

Phylogenetic analysis revealed that strain LP10266 was allied with *L. casei* and *L. paracasei*. Genomic studies revealed two spaCBA pilus clusters and one novel exopolysaccharides (EPS) cluster in strain LP10266, which was sensitive to ampicillin, penicillin, levofloxacin, and imipenem, but resistant to cefuroxime, cefazolin, cefotaxime, meropenem, and vancomycin. Strain LP10266 was nonfatal and sensitive to serum, capable of activating complement 3a and terminal complement complex C5b-9 (TCC). Strain LP10266 could not induce platelet aggregation but displayed a stronger biofilm formation ability and adherence to human vascular endothelial cells (HUVECs) compared to the standard control strain *L. paracasei* ATCC25302.

**Conclusion:**

The genome of a novel bacterial strain *L. paracasei* LP10266 was sequenced. Our results based on various types of assays consistently revealed that *L. paracasei* LP10266 was a potential pathogen to patients with a history of cardiac disease and inguinal hernia repair. Strain LP10266 showed strong biofilm formation ability and adherence, enhancing the awareness of *L. paracasei* infections.

**Supplementary Information:**

The online version contains supplementary material available at 10.1186/s12866-021-02253-8.

## Background

Lactobacilli are Gram-positive, microaerophilic, or facultatively anaerobic, non-spore-forming rods [1]. They form a part of the normal human microbiota of the oral, gastrointestinal, and female genital tracts [2, 3]. They are well-recognized probiotics that help humans enhance immunity [4]. Lactobacilli are not only widely used in many food fermentation processes [5], but also are used for the prevention and treatment of diverse types of intestinal infections caused by pathogenic bacteria [6].

Although lactobacilli are widely used as probiotics, occasionally they act as pathogens to humans [7]. Recently, an increasing number of studies have reported clinical infections caused by lactobacilli*,* including peritonitis, bacteraemia, and endocarditis [8–10]. These infections are attributable to defective host defense mechanisms and severe underlying diseases. The mortality of endocarditis associated with *Lactobacillus* is as high as 30% [11]. Therefore, exploring the potential pathogenic mechanism of lactobacilli is necessary in order to find appropriate treatments to the patients. Previous studies have primarily focused on the complement-mediated immune escape [12] of lactobacilli and their ability to aggregate human platelets [13]. Bacterial biofilm formation [14] and adhesion to human vascular endothelial cells (HUVECs) [15] are also potential pathogenic traits for the progression of infective endocarditis (IE).

Herein, we collected a *Lactobacillus paracasei* isolate from an IE patient’s blood and marrow samples. We further identified this isolate as a novel strain *L. paracasei* LP10266 based on Vitek MS, *16S rRNA* polymerase chain reaction (PCR), and biochemical tests. High-throughput sequencing technology [16] was used to predict the tissue invasion and identify biomarkers implicated in the pathogenicity of lactobacilli [17]. Our results have demonstrated that *L. paracasei* LP10266 has shown shared pathogenic properties with other pathogenic bacteria and evolved multiple mechanisms to adapt and survive in human body.

We conducted a comprehensive assessment of the bacterial pathogenicity and infection of strain LP10266 both in vivo and in vitro, including (1) the virulence by mouse lethality and serum bactericidal assays, (2) the serum complement- and platelet-activating ability, and (3) the biofilm formation and adherence.

## Results

### Case report

A 47-year-old male was admitted to Shandong provincial hospital with a 2-month history of fever, headache, myalgia, arthralgia, and malaise. Before admission, he had been treated with cefoperazone schubatam and tetracycline for a week. However, his fever improved only marginally, while overexertion, palpitation, and dyspnoea were insidious and gradually progressed. The patient had a history of inguinal hernia and a repair surgery 10 years ago. He denied the presence of any chest pain, abdominal pain, nausea, vomiting, or diarrhea, but admitted a history of cardiac disease. On admission, his body temperature was 37.3 °C. Physical examination revealed the aortic valve diastolic murmurs at the precordium. Laboratory results revealed hypoalbuminemia and infection, as indicated by white blood cell count of 10.36 × 10^3^ cells/μl (78.2% neutrophils), C-reactive protein level of 40.14 mg/l, serum ferritin level of 422 ng/ml, procalcitonin level of 0.09 ng/ml, erythrocyte sedimentation rate of 37 mm/h, albumin level of 2.3 g/dl, immunoglobulin E level of 366 IU/ml, complement C4 level of 0.51 g/l, complement C1q level of 0.296 g/l, and N-terminal pro-brain natriuretic peptide level of 532.60 pg/ml.

**Fig. 1:**
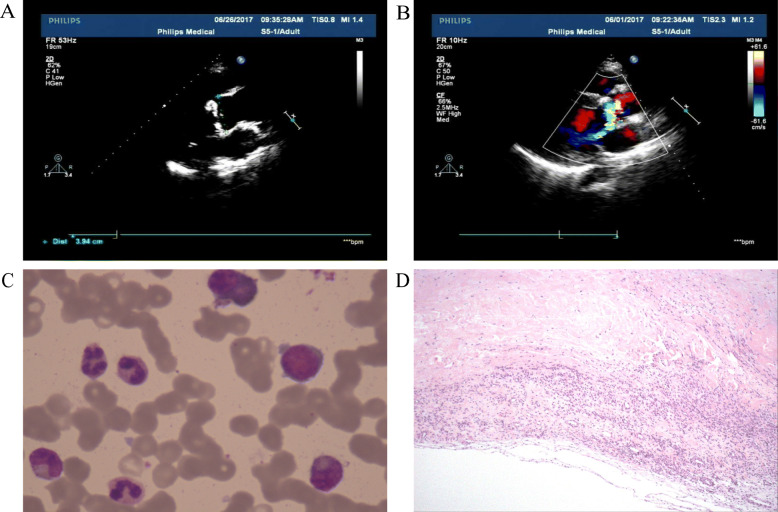
Results of transthoracic echo-cardiogram and stained blood and marrow samples. Marrow and aortic valve excrescence samples collected from the patient on admission showing the growth of Gram-positive rods after cultured on 5% sheep blood agar and incubated at 37 °C in an anaerobic chamber with 5% CO2 for 48 h. A) The membranous ventricular septum bulged into the right ventricle. B) Aortic valve prolapse and aortic valve excrescence formation combined with aortic valve regurgitation at moderate level. C) Wright-Giemsa staining of bone marrow showing infective myelogram. D) Hematoxylin-Eosin staining of cardiac excrescence showing infectious excrescence

The transthoracic echocardiogram showed a left ventricular ejection fraction of 50% and membranous ventricular septal bulging into the right ventricle (Fig. 1). Aortic valve prolapse and aortic valve excrescence formation combined with aortic valve regurgitation at moderate level were found. Marrow and aortic valve excrescence samples collected from the patient on admission showed the growth of Gram-positive rods after being cultured on 5% sheep blood agar and incubated at 37 °C in an anaerobic chamber with 5% CO_2_ for 48 h (Fig. 1).

The patient was initially treated with ceftriaxone (2 g i.v. every 24 h) and cefoperazone schubatam (3 g i.v. every 12 h) for 4 days, resulting slight clinical improvement and temperature raise to 38.6 °C on the fifth day. Antibiotic susceptibility tests showed that *L. paracasei* LP10266 was resistant to cefuroxime, cefazolin, cefotaxime, meropenem, and vancomycin, but susceptible to ampicillin, penicillin, clindamycin, levofloxacin, and imipenem, suggesting that the empirical therapy targeting common Gram-positive bacterium might fail in the treatment. The patient was then treated with tazobactam/piperacillin (2.25 g i.v. every 12 h) after the isolate was identified as *L. paracasei* on day 5. The results of blood culture became negative on day 20. The patient underwent an aortic valve replacement with a mechanical prosthesis after 3 weeks of antibiotic treatment and the blood cultures became sterile.

### Bacterial identification and susceptibility

The isolates from the patient’s blood were cultured on 5% sheep blood agar and incubated at 37 °C in an anaerobic chamber with 5% CO_2_ for 48 h. The bacterial colonies obtained were small (2-3 mm in diameter), non-haemolytic, convex, and smooth along the entire edge. The curved Gram -positive rods were identified as a novel strain *L. paracasei* LP10266 based on Vitek MS (bioMérieux, France), *16S rRNA* polymerase chain reaction (PCR), and biochemical tests. Antimicrobial susceptibility testing was performed by the microdilution broth method using CAMHB-LHB, according to Clinical & Laboratory Standards Institute (CLSI) guidelines. Results of antibiotic susceptibility testing showed that the strain LP10266 was resistant to cefuroxime, cefazolin, ceftriaxone, meropenem, and vancomycin, and susceptible to ampicillin, penicillin, levofloxacin, and imipenem (Table 1).

**Table 1:** Antibiotic susceptibilities of *L. paracasei* LP10266

Antibiotics	Minimal inhibitory concentrations (μg/ml)
Ampicillin	1.5
Penicillin	0.5
Cefazolin	> 256
Cefuroxime	> 256
Ceftriaxone	> 256
Meropenem	16
Imipenem	1
Vancomycin	> 256
Levofloxacin	1

### Prophage insertion and CRISPR region of *L. paracasei* LP10266

The genome of *L. paracasei* LP10266 was sequenced using PacBio RS II sequencing platform (Menlo Park, USA) and assembled using HGAP 3.0 with default parameters. The complete genome of strain LP10266 contained a circular chromosome of 3.01 Mb in length with the G + C content of 43.362% (Table 2).

**Table 2:** Genome characteristics of *L. paracasei* LP10266

Characteristics	Chromosome	Plasmid
No. of scaffolds	1	1
Bases in all scaffolds (bp)	3,012,260	32,648
No. of large scaffolds (> 1000 bp)	1	1
Bases in large scaffolds (bp)	3,012,260	32,648
Largest length (bp)	3,012,260	32,648
Scaffold N50 (bp)	3,012,260	32,648
Scaffold N90 (bp)	3,012,260	32,648
G + C content (%)	43.362	42.915
N rate (%)	0	0

The prophages A (37.3 kb) and B (47.1 kb) were identified in the genome of LP10266 at nucleotide positions 536,722-574,063 and 1,183,595-1,230,784, respectively. Prophage A was similar to the temperate bacteriophage Lactob_phiAT3 isolated from *L. casei* ATCC393, while the prophage B was similar to the temperate bacteriophage Lactob_PLE3 isolated from the probiotic strain *L. casei* PLE3. Both prophages A and B displayed a mosaic architecture and possessed sequences homologous to phages or strains of *Lactococcus*, *Streptococcus*, *Listeria,* and *Lactobacillus*.

As parts of the distinct defense system in bacterial genomes, CRISPRs and CRISPR-associated genes efficiently cleave foreign DNA (e.g., phages or plasmids) entering the bacterial cells. A CRISPR region was identified in the genome of LP10266 at nucleotide positions 2,298,816-2,300,897 with 31 spacer sequences of 30 and 36 bp in length.

### Phylogenetic analysis

**Fig. 2:**
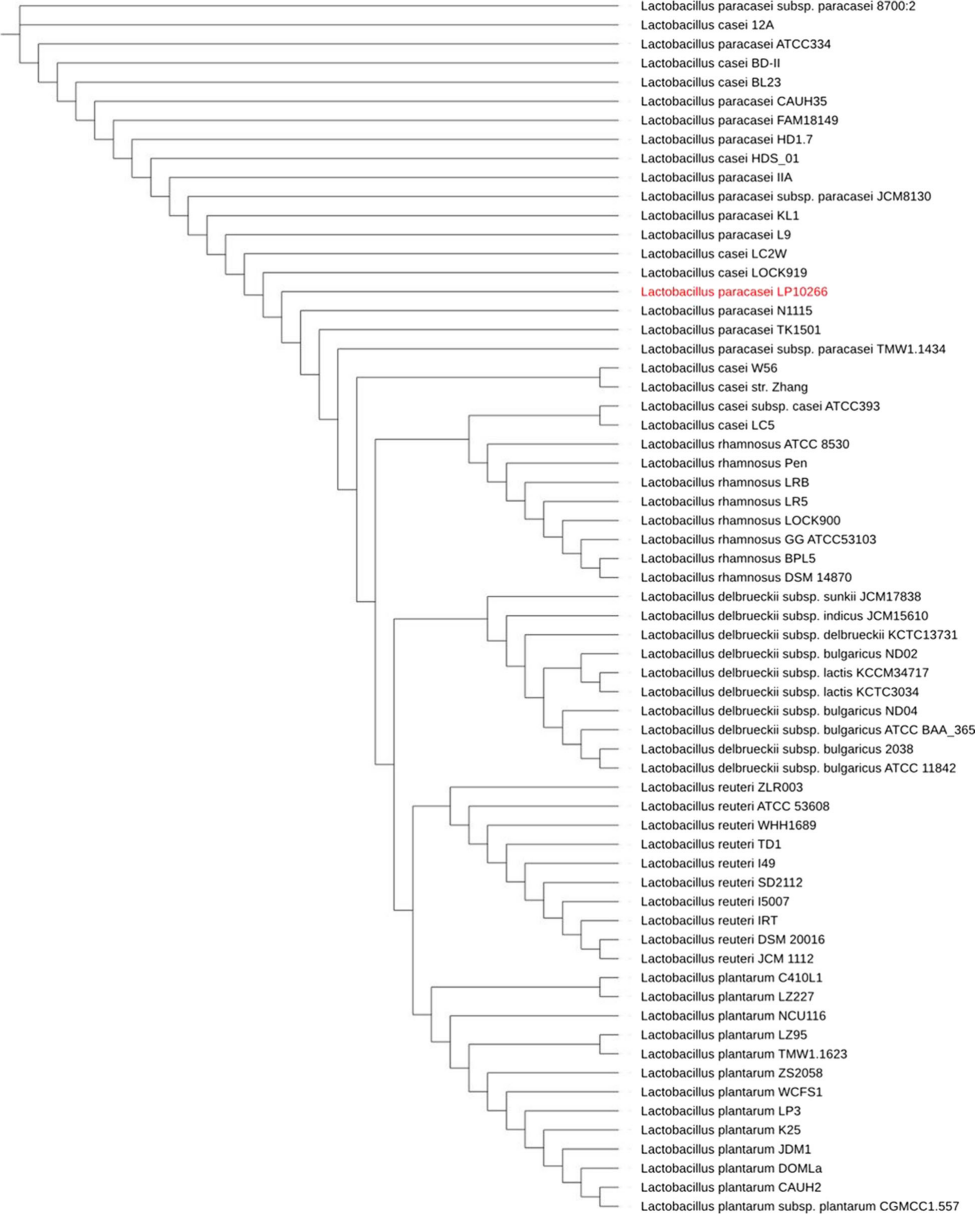
Phylogenetic tree based on the core genomes of 64 strains of lactobacilli. The *L. paracasei* LP10266 is highlighted in red

Comparative analysis revealed high homologous similarities, ranging from 94 to 100%, in the *16S rRNA* gene among the lactobacilli strains. Consequently, we compared the complete genomes of a total of 63 lactobacilli strains with that of strain LP10266 and identified the genetic distances between the core genomes using the unweighted pair group method with arithmetic mean (UPGMA). Phylogenetic analysis revealed that *L. paracasei* LP10266 was allied with *L. casei* and *L. paracasei,* including strains of *L. casei* LC2W, *L. casei* LOCK919, *L. paracasei* N1115, *L. paracasei* TK1501, *and L. paracasei* TMW1.1434 (Fig. 2).

### Pan-genome analysis and unique genes of *L. paracasei* LP10266

**Fig. 3:**
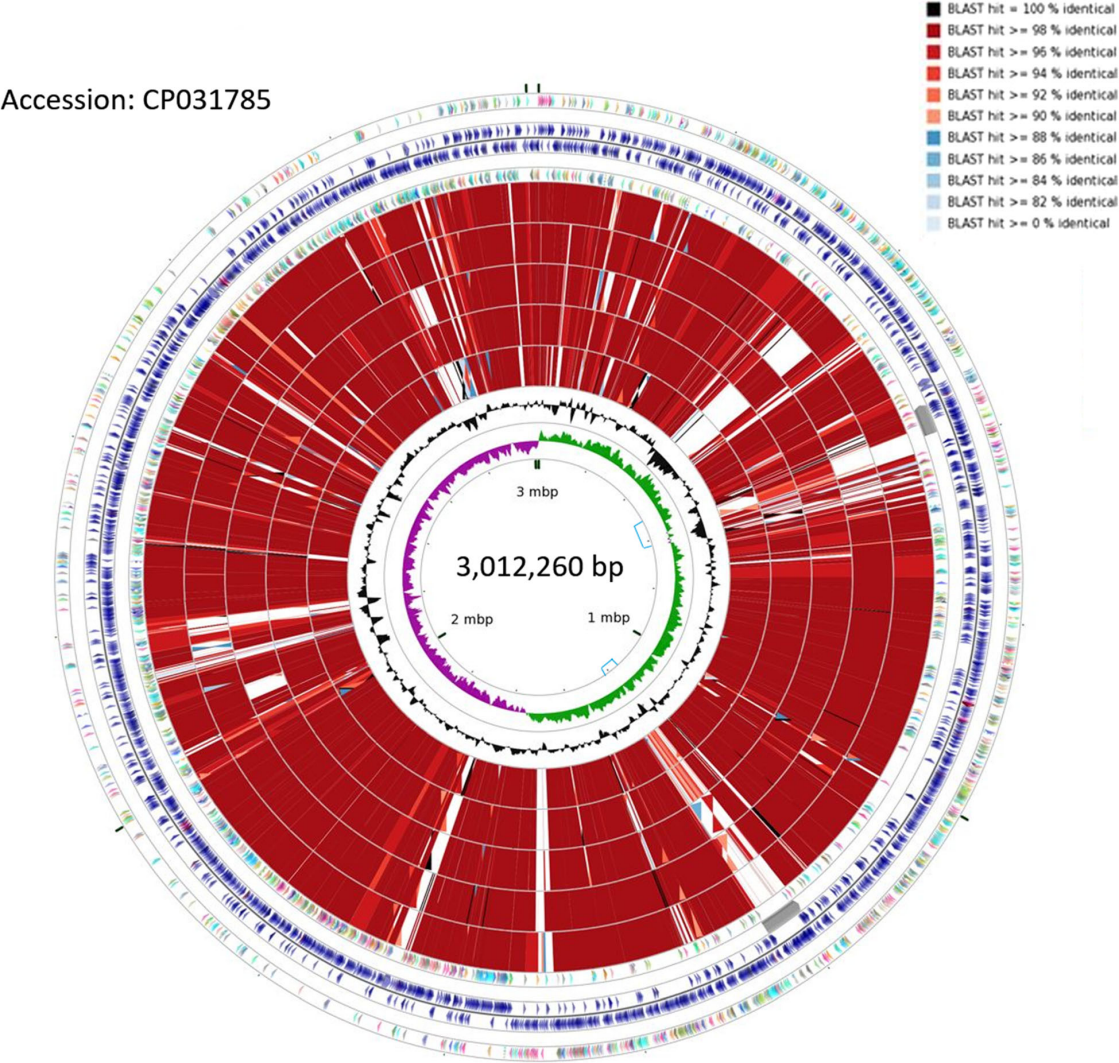
Structural comparison between *L. paracasei* LP10266 and five closely related strains (*L. casei* LC2W, *L. casei* LOCK919, *L. paracasei* N1115, *L. paracasei* TK1501, and *L. paracasei* TMW1.1434) at the genomic level. The rings (from outside to inside) represent CDS annotations on the positive strand, ORFs on the positive and negative strands, and CDS annotations on the negative strand, respectively. The red rings (from outside to inside) represent the BLAST results of *L. paracasei* TMW1.1434 (CP016355), *L. paracasei* TK1501 (CP017716), *L. casei* LC2W (CP002616), *L. casei* LOCK919 (CP005486), and *L. paracasei* N1115 (CP007122). The blue lines framed the prophage sequences at the nucleotide positions 575–618 and 1183–1227, respectively

**Fig. 4:**
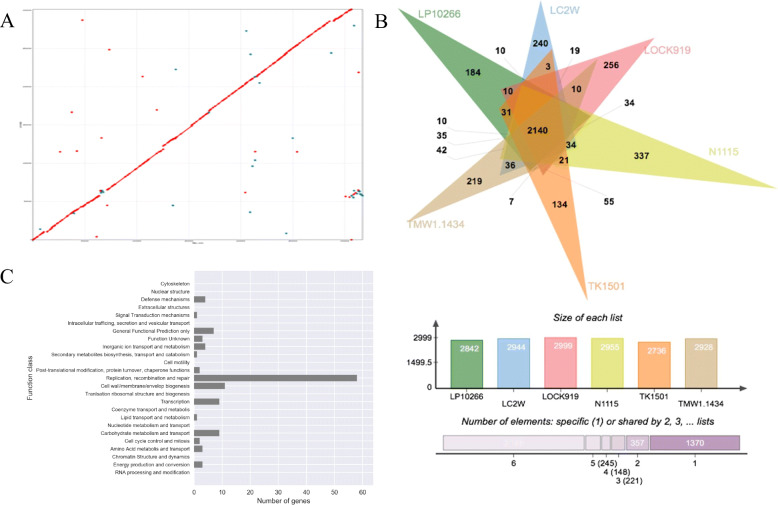
Pan-genome analysis and unique genes of *L. paracasei* LP10266. A) Collinearity analysis between *L. paracasei* LP10266 (vertical axis) and *L. paracasei* TMW1.1434 (horizontal axis). The dots represent the fragments compared in the two genomes. B) Homologous gene analysis using PanOCT showing the numbers of genes in the genome of each bacterial strain. C) COG functional annotation of 110 unique genes in *L. paracasei* LP10266

The closely related six strains of *L. casei* and *L. paracasei* revealed in the phylogenetic tree were further analyzed to infer their genomic structures. The results were visualized using the CGView (Fig. 3). Results showed that the main differences among these genomes were almost consistently revealed in the prophage sequences located at nucleotide positions 575–618 and 1183–1227 (Fig. 3). The best collinearity was identified between the genomes of *L. paracasei* LP10266 and *L. paracasei* TMW1.1434 (Fig. 4A). The numbers of common and unique genes among the six strains were presented in Fig. 4B. A total of 184 out of 2842 genes were exclusively detected in the genome of *L. paracasei* LP10266 compared to the other five strains. Results of COG functional annotation showed that 110 of the 184 unique genes were previously annotated (Fig. 4C).

### Extracellular components and exopolysaccharides (EPS) clusters

To verify the presence of the spaCBA and spaFED clusters in the genome of *L. paracasei* LP10266, we obtained the genome sequence of *L. rhamnosus* ATCC53103 from NCBI (AP011548.1) and extracted three spaCBA protein sequences (cell surface proteins SpaA-LRHM_0426, SpaB-LRHM_0427, and SpaC-LRHM_0428) and three spaFED protein sequences (putative cell surface proteins SpaD-LRHM_2279, SpaE-LRHM_2280, and SpaF-LRHM_2281). Genomic comparison between the genomes of *L. paracasei* LP10266 genome and *L. rhamnosus* ATCC53103 based on BLAST analysis revealed two spaCBA systems but the absence of the spaFED system in the genome of *L. paracasei* LP10266 (Table 3).

**Table 3:** Comparison of the spaCBA system in *L. rhamnosus* ATCC53103 and *L. paracasei* LP10266

***L. rhamnosus*** ATCC53103	***L. paracasei*** LP10266
LRHM_0426 = cell surface protein SpaA	LP10266_00601 Cna protein B-type domain protein LP10266_00499 Cna protein B-type domain protein
LRHM_0427 = cell surface protein SpaB	LP10266_00600 hypothetical protein LP10266_00498 hypothetical protein
LRHM_0428 = cell surface protein SpaC	LP10266_00599 von Willebrand factor type A domain protein LP10266_00497 von Willebrand factor type A domain protein

**Fig. 5:**
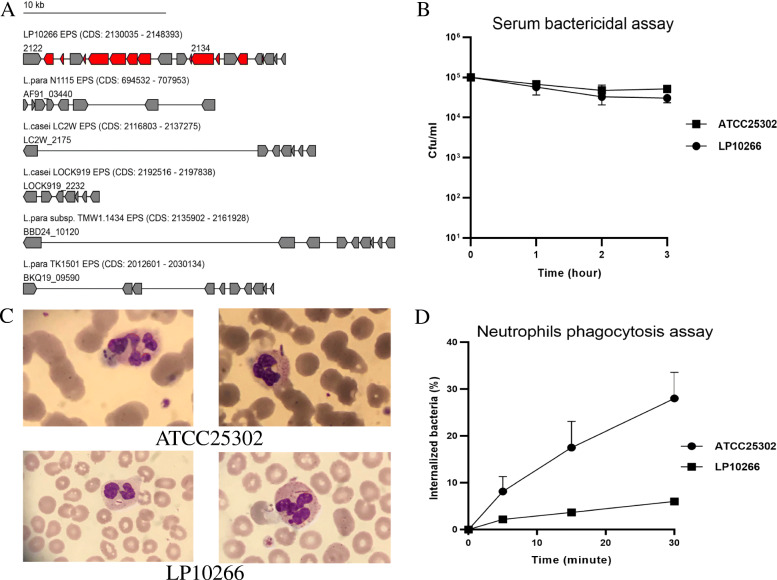
EPS clusters analysis, serum bactericidal assay, and neutrophil phagocytotic assay. A) Genomic organization of the EPS gene cluster in *L. paracasei* LP10266, *L. casei* LC2W, *L. casei* LOCK919, *L. paracasei* N1115, *L. paracasei* TK1501, and *L. paracasei* TMW1.1434. The genes common in all genomes are shown in gray, and the genes exclusively identfied in *L. paracasei* LP10266 are shown in red; 10 kb is the unit length of a gene cluster; the arrow length is proportional to the length of the gene; and the arrow direction represents the transcription direction of the gene. B) Serum bactericidal assay of *L. paracasei* LP10266 and *L. paracasei* ATCC25302. A slight decrease was observed in the growth of *L. paracasei* LP10266. Data are presented as mean ± standard error of mean. C) Microscopic observations under an oil immersion lens of neutrophil phagocytosis of *L. paracasei* ATCC25302 and *L. paracasei* LP10266. D) The correlation between repeated measurements revealed by the test of sphericity (*p* < 0.01). Statistically significant differences were observed at different time points in the overall mean of the data (*p* < 0.01), and interaction was observed between the grouping factors and the repeated measurement factors. The test of Between-Subjects Effects revealed a significant difference of neutrophil phagocytosis between *L. paracasei* strains LP10266 and ATCC25302 (*p =* 0.044)

BLAST analysis of *L. paracasei* LP10266 genome with the EPS-b region in *L. paracasei* strain DG (CNCM I-1572; accession number LT629195) revealed that the region of LP10266_02122-LP10266_02142 was a potential EPS cluster of *L. paracasei* LP10266. This region contained several EPS genes homologous to those of the other five strains. The genomic structures of some of these EPS genes unique to *L. paracasei* LP10266 were further characterized (Fig. 5A).

### Mouse lethality assay, serum bactericidal assay, and neutrophil phagocytic assay

All mice in the two experimental groups remained alive throughout the entire experimental period of 14 days. Each mouse was administered with a single dose of 50 μl of 10^10^ CFU/ml bacterial suspension by caudal vein injection in the mouse lethality assay of *L. paracasei* LP10266 and *L. paracasei* ATCC25302. Following the caudal vein injection of the mice with both strains, the mice were observed for mortality for 14 days. All mice were alive after observation for 14 days on the groups of *L. paracasei* LP10266 and *L. paracasei* ATCC25302. Results showed that *L. paracasei* LP10266 was sensitive to serum (grade 2) (Fig. 5B), while the difference of the neutrophil phagocytic rates of *L. paracasei* LP10266 and *L. paracasei* ATCC25302 were statistically significant (*p* = 0.044) (Fig. 5C, D).

### Complement activity and platelet aggregometry

Both strains of *L. paracasei* LP10266 and *L. paracasei* ATCC25302 could activate C3a (*p* < 0.05) compared with negative controls (with equal volume of 0.9% saline solution), while no difference was observed in the degree of activation between strains ATCC25302 and LP10266 (*p* = 0.216). Formation of the terminal complement complex C5b-9 (TCC) was observed in strain LP10266 (*p* < 0.05) compared with negative controls, while no difference was observed in the degree of activation between strain ATCC25302 and negative controls (*p* = 0.072). Neither LP10266 nor ATCC25302 induced platelet aggregation (Table 4).

**Table 4:** Results of complement 3a (C3a), TCC activity, and platelet aggregometry in *L. paracasei* strains LP10266 and ATCC25302

Variable	LP10266	ATCC25302	Controls	
			NC	ADP
C3a (g/l)	0.207 ± 0.002	0.207 ± 0.014	0.170 ± 0.009	–
TCC (g/l)	0.708 ± 0.036	0.686 ± 0.054	0.581 ± 0.023	–
Platelet aggregation (%)	0	0	9.06 ± 0.017	75.23 ± 0.403

Combined data represent mean ± standard error of mean or numbers of the strains detected. *NC* negative control containing buffer in the assays, *ADP* adenosine diphosphate. Symbol “—” indicates test not available

### Biofilm formation and adhesion to HUVECs

**Fig. 6:**
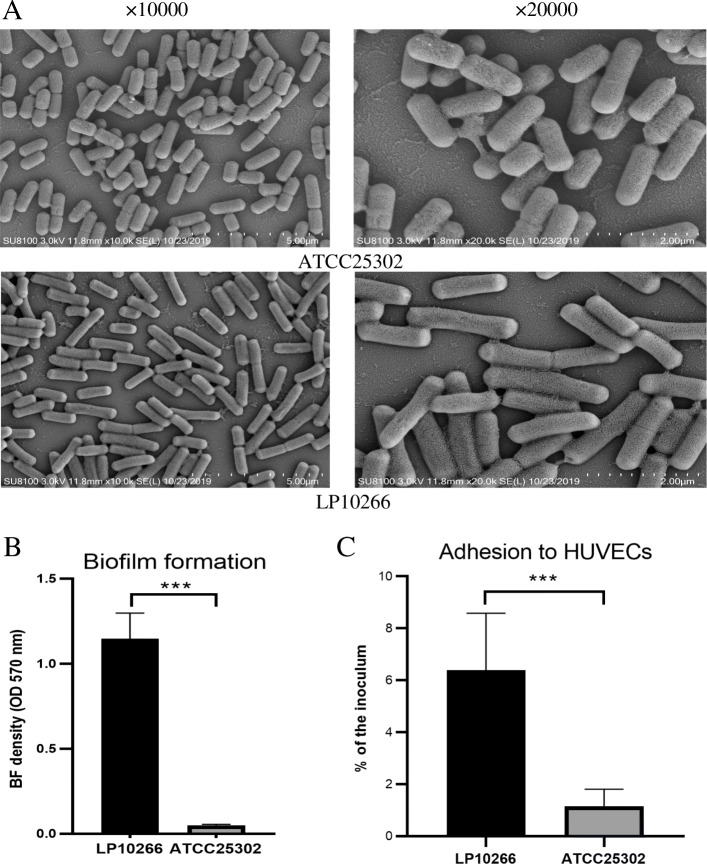
Biofilm formation and adhesion to HUVECs by *L. paracasei* strains ATCC25302 and LP10266. A) Scanning electron microscopic images of *L. paracasei* ATCC25302 and *L. paracasei* LP10266. B) Representation of the results of semi-quantitative adherence assay performed in 96-well polystyrene microtiter plates. Symbols “***” indicate the statistically significant difference between *L. paracasei* ATCC25302 and *L. paracasei* LP10266 (*p* < 0.001). C) Adherence of *L. paracasei* LP10266 and *L. paracasei* ATCC25302 to HUVECs. Data represent the percentages of adherent cells of the initial bacterial inoculum. Symbles “***” indicate the statistically significant difference between *L. paracasei* LP10266 and *L.paracasei* ATCC25302 (*p* < 0.001). Data are presented as the mean ± standard error of the mean of three independent experiments

Microcolony accumulation was observed in both strains of LP10266 and ATCC25302 under scanning electron microscopy (Fig. 6A). The microcolonies produced by strains of LP10266 and ATCC25302 overlapped with each other, forming dense clumps. The results of the biofilm assay revealed a stronger biofilm formation ability in strain LP10266 compared to that of strain ATCC25302 (*p* < 0.001) (Fig. 6B). The results of adhesion to HUVECs revealed that *L. paracasei* LP10266 was more adhesive than *L. paracasei* ATCC25302 (*p* < 0.001) (Fig. 6C).

## Discussion

To the best of our knowledge, this is the first study based on the whole-genome comparative analysis of a *L. paracasei* strain isolated from an IE patient conducted to comprehensively assess the pathogenicity both in vivo and in vitro*.* Generally, lactobacilli isolated from blood are overlooked due to their low virulence perceived [18]. Endocarditis induced by *L. paracasei* is relatively rare [19, 20]. Concerning the antibiotic resistance of lactobacilli, vancomycin and ciprofoxacin show low inhibitory effects among the majority of *Lactobacillus* species [21, 22], which are generally sensitive to chloramphenicol, macrolides, lincosamides, and tetracycline [21, 23, 24]. Moreover, some lactobacilli are resistant to cephalosporins [21, 25]. The cell wall impermeability has been proposed to be a possible mechanism explaining the resistance of some lactobacilli to cephalosporins. Other nonspecific mechanisms are involved with multidrug transporters and defective cell wall autolytic systems [7]. Currently, no evidence or studies are available on *Lactobacillus* suggesting the transferability of resistance genes for β-lactam antibiotics [26]. In the reported case of our study, we suspected the existence of β-lactam resistance genes in *L. paracasei* LP10266. However, no known β-lactam resistance genes were identified and the mechanism of antibiotic resistance remains unknown. The patient underwent an aortic valve replacement with a mechanical prosthesis after 3 weeks of antibiotic treatment of tazobactam/piperacillin (2.25 g i.v. every 12 h) and then the blood cultures became sterile.

Our pan-genome analysis revealed that the unique genes of *L. paracasei* LP10266 are mainly associated with its ability to adapt to new environments through replication, recombination, and repair of its genetic materials. These findings explain the survival and reproduction of *L. paracasei* LP10266 despite its translocation from the intestine to the blood, leading to bacteraemia. However, it has been challenging to elucidate the explicit mechanisms underlying this translocation. Previous studies showed that a large number of patients (64%) were valvulopathy, while ~ 30% of patients had dental procedures or invasive procedures and immunosuppression was only accounted for nearly 20% [27]. The history of cardiac disease and inguinal hernia repair might be the potential risk factors for endocarditis of the patient in our study.

As a facultatively heterofermentative lactobacilli, *L. paracasei* has been usually used for Cheese production and probiotic products [28]. Newly identified probiotic strains might show potential risks to humans. Thus, they are subjected to comprehensive safety and toxicity tests based on the mouse model and serum bactericidal assay in vitro [29]. Strain *L. paracasei* LP10266 was isolated from a patient without typical risk indications of severe immune compromise or enterobrosis, leading us to question the safety of *L. paracasei* LP10266. The mouse lethality assay and serum bactericidal assay revealed that the strain LP10266 was nonfatal and sensitive to serum. Studies have shown that complement receptor-mediated immune evasion caused the persistent bacteremia [30]. However, our results showed that *L. paracasei* LP10266 could effectively activate the complement 3a and TCC.

We further investigated the survival of *L. paracasei* LP10266 in the bloodstream and its possible recognition or destruction by the host immune system. The neutrophil phagocytic assay showed that the difference of the neutrophil phagocytic rate of *L. paracasei* LP10266 and *L. paracasei* ATCC25302 was statistically significant (*p* = 0.044). Platelet aggregation caused by bacteria is an important source of infective endocarditis [31]. Many Gram-positive bacteria causing sepsis have been shown to induce platelet aggregation [32]. However, our experimental data revealed that the blood isolate of *L. paracasei* LP10266 did not cause platelet aggregation.

Furthermore, the binding of lactobacilli to the heart valve may be of importance in the early stages of colonization. The Genomic analysis revealed spaCBA pilus gene cluster and EPS cluster in *L. paracasei* LP10266. The spaCBA protein complex is a heterotrimeric pilus, which functions as an adhesive protein in lactobacilli, as confirmed for the first time in *L. rhamnosus* GG [33]. The spaCBA not only functions as an adhesion molecule but is also involved in the formation of a biofilm [34]. However, some bacterial strains containing intact spaCBA coding sequence exhibited weak adhesion efficiency [35]. EPS has been reported to be involved in biofilm formation, showing adhesive properties and immunomodulation by probiotic strains [36]. In a previous study, EPS was involved in the lactobacilli-host interactions, especially with the intestinal mucosa and epithelial cells, thus contributing to the strain-specific probiotic characteristics [37]. Therefore, we speculated that biofilm formation and the binding of *L. paracasei* LP10266 to the heart valve may be critical for bacterial survival in blood and excrescence formation on the heart valves. Our findings indicate strong adherence ability and biofilm formation of *L. paracasei* LP10266, providing strong evidence to help us explore the underlying mechanisms involved in IE caused by strain LP10266.

In conclusion, our results indicated that, as a pathogen, strain LP10266 is limited by its low virulence and ease of being killed by serum. Although *L. paracasei* LP10266 was resistant to vancomycin, cefuroxime, cefazolin, cefotaxime, and meropenem, the patient was cured by the treatment of tazobactam/piperacillin. The invasions of *L. paracasei* LP10266 and ATCC25302 showed no difference, while LP10266 showed stronger ability of biofilm formation and adhesion than ATCC25302*.* Furthermore, our results showed that *L.paracasei* LP10266 contained the spaCBA pilus gene cluster and EPS cluster which are highly associated with biofilm formation and adherence, as demonstrated by adhesion to HUVECs and biofilm formation assays. Therefore, strain LP10266 was a conditional pathogen to patients with a history of cardiac disease and inguinal hernia repair. We note that the limitation of our study is that we did not further investigate the underlying genetic mechanisms regulating the potential assocations between both the gene clusters of spaCBA pilus and EPS with IE. Future genetic knock-out experiments are required to verify these associations. With the available genome of strain LP10266, we foresee the identification of the potential genes in LP10266 involved in the formation of IE.

## Conclusions

The present study reported a rare case of infective endocarditis caused by a novel bacterial strain of *L. paracasei* LP10266. Comparative genomic analysis revealed that strain LP10266 was closely related with *L. casei* and *L. paracasei*. Two prophage insertions and one CRISPR region were identified in the genome of *L. paracasei* LP10266. Furthermore, the spaCBA pilus gene cluster and EPS cluster (LP10266_02122-LP10266_02142) were identified in *L. paracasei* LP10266. Our results indicated that *L. paracasei* LP10266 exhibited low virulence but strong ability of biofilm formation and adhesion. Moreover, strain LP10266 was a conditional pathogen to patients with a history of cardiac disease and inguinal hernia repair. The data presented in this study provided the essential genetic foundation for further comprehensive investigation of the pathogenic mechanisms of this clinically important bacterial strain of *L. paracasei*.

## Materials and methods

### Growth conditions and antimicrobial susceptibility testing of bacterial isolates

The bacterial isolates from the IE patient’s blood were cultured on 5% sheep blood agar and incubated at 37 °C in an anaerobic chamber with 5% CO_2_ for 48 h. The curved Gram-positive rods, identified using Vitek MS (bioMérieux, France), *16S rRNA* polymerase chain reaction (PCR), and biochemical tests, was named *L. paracasei* LP10266. The *L. paracasei* reference stain ATCC25302 was retrieved from the China General Microbiological Culture Collection Center (http://www.cgmcc.net/english/).

Lactobacilli were purified and cultured anaerobically in de Man, Rogosa, and Sharpe (MRS) broth (Hopebiol, China) at 37 °C for 20 h. Antimicrobial susceptibility testing was performed by the microdilution broth method using CAMHB-LHB, according to Clinical & Laboratory Standards Institute (CLSI) guidelines.

### Genome sequencing and gene annotation

Genomic DNA was extracted and purified using the QIAamp DNA Mini Kit (Qiagen, Germany). The genome was sequenced on the PacBio RS II sequencing platform (Menlo Park, USA) and assembled using HGAP 3.0 with default parameters. The genome was annotated using the NCBI Prokaryotic Genome Annotation Pipeline (PGAAP). BLASTP analysis of the coding genes was performed against the genes in the Swiss-Prot database, the non-redundant protein database (NR), and the Clusters of Orthologous Groups of proteins (COG) database. Clustered regularly interspaced short palindromic repeats (CRISPRs) were detected using CRISPRFinder [38]. Potential virulence factors were screened using SignalP 4.0 [39] and TMHMM 2.0 [40]. The annotated sequences of *L. paracasei* LP10266 chromosome and plasmids were deposited in the GenBank (https://www.ncbi.nlm.nih.gov/genbank/) with accession numbers CP031785 and CP031786, respectively.

### Comparative genomic analysis

Using the complete genome sequences of a total of 63 *Lactobacillus* species (13 *L. plantarum*, 22 *L. casei*, 10 *L. delbrueckii*, 12 *L. paracasei*, 10 *L. reuteri,* and 7 *L. rhamnosus*) from NCBI (Supplementary Table 1) and *L. paracasei* LP10266, we constructed a pan-genome using the Panseq [41]. We calculated the distances between the core genome sequences obtained using Phylip [42]. Subsequently, a phylogenetic tree was constructed using the unweighted pair group method with arithmetic mean (UPGMA) by PHYLIP. The target strains closely related to each other revealed in the phylogenetic tree were subjected to further genomic structural comparison analysis. The results were visualized using the CGView [43].

Based on the results of genomic structural comparison analysis, the analyses of structural variation (SV), single nucleotide polymorphism (SNP), and insertion and deletion (InDel) in *L. paracasei* LP10266 were assessed based on the LargeScale Genome Alignment Tool (LastZ) [44]. The obtained SNPs were annotated using the gff annotation file.

### Unique genomic features

To further investigate the genomic features of *L. paracasei* LP10266, we constructed a pan-genome using PanOCT v3.23 [45]. The unique genes of *L. paracasei* LP10266 were identified and annotated.

### Exopolysaccharide (EPS) cluster analysis

The EPS cluster in the genome of *L. paracasei* LP10266 was subjected to BLAST analysis with an e-value <1e-10. Subsequently, the obtained EPS cluster was BLASTed against the genomes of the other five *Lactobacillus* strains (*L. casei* LC2W, *L. casei* LOCK919, *L. paracasei* N1115, *L. paracasei* TK1501, and *L. paracasei* TMW1.1434) with an e-value <1e-10. The obtained clusters were visualized using Perl to identify the genomic differences among the EPS clusters.

### Mouse lethality assay

A total of 18 pathogen-free male BALB/c mice (6–8 weeks old) were evenly divided into 3 groups (one control and two experimental groups) [13] for every test. There was a total of 54 mice. The mice were purchased from the Jinan Pengyue Experimental Animal Breeding Co., Ltd. (Jinan, China), with a qualified number of 370,726,211,100,333,967. Each mouse was administered with a single dose of 50 μl of 10^10^ CFU/ml bacterial suspension by caudal vein injection. The control group received the same volume of sterile 0.9% saline solution. The mice were housed under standard conditions of alternate light and dark cycles (12 h each) at 25 ± 2 °C for 14 days. The mice were sacrificed by CO_2_ inhalation after the observation of 14 days according to the American Veterinary Medical Association (AVMA) Guidelines for the Euthanasia of Animals (2020). Each bacterial strain was tested at least thrice. Data were presented as mean *±* standard error of the mean and the mean was expressed as percentage of inoculum.

### Neutrophil phagocytotic assay

As previously described [46], 100 μl of 10^8^ CFU/ml bacterial suspension was added to 900 μl human sodium citrate anticoagulant in a 1.5 ml EP tube and incubated at 37 °C for 0, 5, 15, and 30 min, respectively. Samples were prepared on slides and stained with Wright-Giemsa stain, and observed a total of 200 neutrophils randomly selected under an oil immersion lens. Subsequently, the number of phagocyted bacterial cells was recorded. The experiment was repeated thrice each with three replicates. The results were presented as mean ± standard error of mean.

### Determination of complement activity using complement 3a and terminal complement complex C5b-9 (TCC) based on ELISA

Approximately 10 ml human blood were collected from a healthy individual and kept in blood collection tubes without any additives. The serum was obtained by centrifugation at 780 g for 20 min. A total of 100 μl of 10^8^ CFU/ml bacterial suspension was mixed with an equal volume of serum and incubated for 30 min at 37 °C, with agitation at 500 rpm, to activate the complement system in the serum of the healthy volunteer. The reactions were terminated by adding EDTA (final concentration of 15 mM) and incubated on ice. Subsequently, the serum samples were diluted to 1:2000, and the complement 3a and TCC in the samples were analysed using the human complement fragment 3a ELISA and TCC kits (Shanghai Fusheng Industrial Co., Ltd., Shanghai, China), respectively, as described previously [47]. All experiments were repeated thrice each with three replicates. The results were presented as mean ± standard error of mean.

### Serum bactericidal assay

Approximately 10 ml human blood were collected from a healthy individual in a blood collection tube without additives and centrifuged at 3130 g for 10 min to obtain the serum. A total of 25 μl of 10^5^ CFU/ml microbial inoculum was added to 75 μl serum in a 1.5 ml EP tube. Subsequently, 10 μl of the mixture were poured evenly on Columbia blood agar plates and incubated for 0, 1, 2, and 3 h, respectively, for colony count. Each bacterial strain was tested at least thrice each with three replicates. The results were presented as mean ± standard error of mean and the mean was expressed as percentage of inoculum. The strains belonging to grades 1-2 were defined as serum sensitive, grades 3-4 as intermediate sensitive, and grades 5-6 as resistant [48]. The *L. paracasei* ATCC25302 was used as the control strain.

### Platelet preparation and aggregometry

Approximately 10 ml of blood samples were collected from healthy donors as described previously [49]. Whole blood samples were centrifuged at 160 g for 10 min at 22 °C. Then, the platelet-rich plasma (PRP) was collected gently using a Pasteur pipette and transferred into separate plastic tubes. Platelet-poor plasma (PPP) was obtained by further centrifuging at 2000 g for 10 min at 22 °C. Platelet aggregation was recorded by an aggregometer. Light transmission through PPP and PRP represented 100 and 0% aggregation, respectively.

Platelet viability was confirmed by the addition of adenosine diphosphate (ADP) as an agonist to PRP with a final concentration of 5 μM. A total of 13 μl 10^9^ CFU/ml bacterial suspension was added to 250 μl PRP. One minute after the addition of ADP agonist or bacteria into the well, the platelet response was monitored for 30 min. Negative aggregation was determined based on the lag phase longer than 25 min of the bacterial growth. All experiments were repeated thrice each with three replicates. The results were presented as mean ± standard error of mean.

### Biofilm assay

A total of 200 μl bacterial culture in MRS medium (dilution = 1:199) kept overnight was added to the wells of a sterile 96-well flat-bottomed plastic tissue culture plate, and the sterile broth was added to the negative control wells. The plates were incubated at 37 °C for 36 h for biofilm formation. Following incubation, the wells were washed with 250 μl PBS buffer thrice. The adherent bacterial biofilms were fixed by adding 200 μl methanol to each well and incubating for 15 min. Subsequently, the plates were emptied and left to dry at room temperature. The wells were then stained with 200 μl 0.1% crystal violet for 5 min. Excess dye was rinsed with water and the wells were air-dried at room temperature. The dye bound to the adherent cells was extracted with 200 μl 30% glacial acetic acid. The optical density (OD) was measured at 570 nm [34]. All experiments were repeated thrice each with eight replicates. The results were presented as mean ± standard error of the mean.

### Scanning electron microscopy

Monoclonal strains were selected from the plate and cultured overnight in MRS medium at 37 °C. Then, the cultures were diluted (1:200) in MRS medium and their OD values were measured using a turbidimeter to obtain cultures with the same initial density (10^8^ CFU/ml). A glass disk was placed at the bottom of a sterile flat-bottomed polystyrene plate (Costar 3524; Corning, NY, USA) with 1 ml of the cell suspension added to each of the wells. The plates were incubated overnight at 37 °C without shaking. Subsequent procedures followed those as described previously [50].

### Adherence assay using HUVECs

One millilitre of suspension containing 10^5^ primary HUVECs (Cat. No. 8000; primary culture cell; Sciencell) was seeded into each well of a 24-well flat-bottomed tissue culture plate coated with poly-L-lysine (Cat. P2100, Solarbio) and incubated overnight at 37 °C in a 5% CO_2_ atmosphere in endothelial cell medium (ECM; Cat. #1001; Sciencell) with penicillin-streptomycin. Prior to infection, the wells were washed thrice with pre-warmed ECM without antibiotics and incubated for 2 h.

As described previously [51–53], lactobacilli were cultured in MRS for 18 h, washed twice with PBS buffer, and re-suspended in antibiotics-free ECM at a concentration of 10^8^ CFU/ml. Subsequently, a total of 10 μl bacterial suspension was added in each well and incubated for 2 h in the absence of antibiotics. The wells were washed thrice with PBS buffer to remove non-adherent bacteria. A total of 500 μl of sterile distilled water was added and incubated aerobically at 37 °C for 20 min. Dilutions of the cell lysates infected with bacteria were plated in MRS agar and cultured at 37 °C for 24 h.

The adhesion was calculated as the percentage of bacteria adhering to HUVECs over the total number of bacteria added to the wells [15]. All experiments were repeated thrice each with three replicates. The results were presented as mean ± standard error of mean.

### Statistical analysis

All Statistical analyses were performed with SPSS™ software version 22.0 (IBM Corporation, Armonk, NY, USA). A *p* value < 0.05 was considered statistically significant.

## Supplementary Information

**Additional file 1: Supplementary Table 1.** Genomic characterization statistics of 63 *Lactobacillus* species from NCBI.

## Data Availability

The datasets generated and/or analysed during the current study are available in the GenBank (https://www.ncbi.nlm.nih.gov/genbank/) repository, [ACCESSION NUMBER: CP031785 and CP031786].

## Abbreviations

IE: Infective endocarditis
EPS: Exopolysaccharides
TCC: Terminal complement complex C5b-9
HUVECs: Human vascular endothelial cells
PCR: Polymerase chain reaction
CLSI: Clinical & Laboratory Standards Institute
Mb: Megabase pair
kb: Kilobase pair
bp: Base pair
CRISPRs: Clustered regularly interspaced short palindromic repeats
CDS: Coding sequence
BLAST: Basic Local Alignment Search tool
NCBI: National Center for Biotechnology Information
ATCC: American Type Culture Collection
CFU: Colony-forming units
PBS: Phosphate buffer saline
