# Supplementary material for: Characterization of a bacterial strain Lactobacillus paracasei LP10266 recovered from an endocarditis patient in Shandong, China
Source: BMC Microbiol. 2021 Jun 17;21:183. doi: 10.1186/s12866-021-02253-8 (PMC8210379; doi:10.1186/s12866-021-02253-8)
Supplement: Supplementary file 1 — Additional file 1: Supplementary Table 1. Genomic characterization statistics of 63 Lactobacillus species from NCBI. [file 12866_2021_2253_MOESM1_ESM.docx]

**Supplementary Table S1** Genomic characterization statistics of 63 *Lactobacillus* species from NCBI.

| **strain** | **contigs** | **bases** | **rRNA** | **tRNA** | **sig_peptide** | **CDS** | **misc_RNA** | **tmRNA** |
| --- | --- | --- | --- | --- | --- | --- | --- | --- |
| FAM18149 | 6 | 2,969,707 | 15 | 59 | 136 | 2,968 | 53 | 1 |
| LC5 | 1 | 3,132,867 | 15 | 60 | 159 | 2,912 | 48 | 1 |
| ZS2058 | 1 | 3,198,337 | 15 | 64 | 161 | 2,953 | 62 | 1 |
| C410L1 | 7 | 3,392,777 | 16 | 70 | 166 | 3,243 | 68 | 1 |
| JCM1112 | 1 | 2,039,414 | 18 | 65 | 68 | 2,020 | 71 | 1 |
| LOCK900 | 1 | 2,883,376 | 15 | 59 | 133 | 2,650 | 48 | 1 |
| KCTC3034 | 1 | 2,237,608 | 27 | 94 | 107 | 2,143 | 39 | 1 |
| Pen | 1 | 2,884,966 | 15 | 59 | 133 | 2,666 | 48 | 1 |
| CGMCC1 | 3 | 3,273,239 | 16 | 71 | 152 | 3,097 | 65 | 1 |
| ATCC8530 | 1 | 2,960,339 | 15 | 61 | 149 | 2,726 | 48 | 1 |
| ND02 | 2 | 2,131,976 | 27 | 98 | 113 | 2,039 | 37 | 1 |
| TD1 | 1 | 2,145,445 | 18 | 71 | 70 | 1,981 | 79 | 1 |
| BPL5 | 1 | 3,024,027 | 15 | 61 | 151 | 2,814 | 47 | 1 |
| ATCC53608 | 3 | 2,091,243 | 18 | 75 | 64 | 2,052 | 51 | 2 |
| ND04 | 1 | 1,861,754 | 27 | 95 | 87 | 1,884 | 37 | 1 |
| 12A | 1 | 2,907,892 | 15 | 58 | 165 | 2,743 | 46 | 1 |
| DOMLa | 3 | 3,210,111 | 16 | 64 | 155 | 2,988 | 60 | 1 |
| LC2W | 2 | 3,077,434 | 15 | 59 | 163 | 2,986 | 49 | 1 |
| Zhang | 2 | 2,898,456 | 15 | 60 | 158 | 2,694 | 53 | 1 |
| ZLR003 | 1 | 2,234,097 | 18 | 69 | 68 | 2,176 | 52 | 1 |
| DSM20016 | 1 | 1,999,618 | 18 | 68 | 67 | 1,983 | 69 | 1 |
| LP3 | 3 | 3,329,954 | 16 | 69 | 163 | 3,098 | 64 | 1 |
| KCTC13731 | 1 | 1,910,506 | 24 | 84 | 106 | 1,832 | 35 | 1 |
| GG_ATCC53103 | 1 | 3,010,111 | 15 | 57 | 148 | 2,817 | 49 | 1 |
| NCU116 | 1 | 3,354,689 | 16 | 67 | 168 | 3,149 | 65 | 1 |
| BL23 | 1 | 3,079,196 | 15 | 60 | 165 | 2,996 | 49 | 1 |
| IIA | 4 | 3,246,143 | 15 | 60 | 163 | 3,134 | 53 | 1 |
| KL1 | 1 | 2,918,888 | 15 | 60 | 152 | 2,846 | 47 | 1 |
| JCM15610 | 3 | 2,021,861 | 27 | 97 | 108 | 1,980 | 34 | 1 |
| LZ227 | 6 | 3,425,292 | 16 | 72 | 164 | 3,297 | 74 | 1 |
| TMW1 | 5 | 3,332,882 | 16 | 70 | 179 | 3,125 | 72 | 1 |
| ATCC393 | 3 | 2,952,961 | 15 | 59 | 130 | 2,890 | 45 | 1 |
| HDS_01 | 1 | 3,038,767 | 15 | 60 | 159 | 2,916 | 49 | 1 |
| 2038 | 1 | 1,872,918 | 27 | 89 | 97 | 1,923 | 36 | 1 |
| N1115 | 5 | 3,064,279 | 15 | 62 | 149 | 3,005 | 51 | 1 |
| WHH1689 | 1 | 2,044,184 | 18 | 69 | 58 | 2,081 | 88 | 1 |
| CAUH35 | 5 | 2,973,347 | 15 | 59 | 142 | 2,946 | 51 | 1 |
| BD_II | 2 | 3,127,288 | 15 | 60 | 168 | 3,066 | 49 | 1 |
| ATCC_BAA365 | 1 | 1,856,951 | 27 | 98 | 97 | 1,906 | 36 | 1 |
| IRT | 1 | 1,993,967 | 18 | 69 | 67 | 1,956 | 71 | 1 |
| K25 | 7 | 3,412,154 | 16 | 69 | 164 | 3,244 | 70 | 1 |
| ATCC11842 | 1 | 1,864,998 | 27 | 95 | 96 | 1,900 | 32 | 1 |
| WCFS1 | 4 | 3,348,624 | 16 | 71 | 165 | 3,123 | 62 | 1 |
| 8700_2 | 3 | 3,025,352 | 15 | 60 | 170 | 2,901 | 49 | 1 |
| L9 | 1 | 3,076,437 | 15 | 59 | 155 | 2,943 | 46 | 1 |
| LRB | 1 | 2,934,954 | 15 | 59 | 143 | 2,880 | 49 | 1 |
| DSM_14870 | 1 | 3,013,149 | 15 | 55 | 145 | 2,789 | 51 | 1 |
| W56 | 2 | 3,132,096 | 15 | 60 | 165 | 3,145 | 50 | 1 |
| HD1 | 1 | 3,039,280 | 15 | 60 | 159 | 2,942 | 49 | 1 |
| KCCM34717 | 1 | 2,263,382 | 32 | 98 | 108 | 2,169 | 41 | 1 |
| I5007 | 7 | 2,093,275 | 18 | 69 | 64 | 2,032 | 51 | 1 |
| JCM8130 | 3 | 3,017,804 | 15 | 62 | 157 | 2,945 | 47 | 1 |
| ATCC334 | 2 | 2,924,325 | 15 | 60 | 151 | 2,835 | 52 | 1 |
| LP10266 | 2 | 3,044,908 | 15 | 60 | 159 | 2,887 | 48 | 1 |
| JCM17838 | 1 | 2,004,337 | 27 | 95 | 119 | 1,853 | 39 | 1 |
| TK1501 | 1 | 2,942,538 | 15 | 59 | 155 | 2,760 | 46 | 1 |
| I49 | 1 | 2,044,771 | 18 | 70 | 81 | 1,894 | 68 | 1 |
| LZ95 | 3 | 3,322,458 | 16 | 71 | 176 | 3,121 | 65 | 1 |
| LR5 | 1 | 2,972,590 | 15 | 60 | 149 | 2,749 | 49 | 1 |
| CAUH2 | 4 | 3,274,625 | 17 | 72 | 174 | 3,056 | 62 | 1 |
| LOCK919 | 2 | 3,143,369 | 15 | 60 | 170 | 3,007 | 48 | 1 |
| JDM1 | 1 | 3,197,759 | 16 | 60 | 155 | 2,980 | 60 | 1 |
| SD2112 | 5 | 2,316,838 | 18 | 70 | 65 | 2,281 | 98 | 1 |
